# Peer Feedback Reflects the Mindset and Academic Motivation of Learners

**DOI:** 10.3389/fpsyg.2020.01701

**Published:** 2020-07-16

**Authors:** Junfeng Zhang, Elina Kuusisto, Petri Nokelainen, Kirsi Tirri

**Affiliations:** ^1^Faculty of Educational Sciences, University of Helsinki, Helsinki, Finland; ^2^Faculty of Education and Culture, Tampere University, Helsinki, Finland

**Keywords:** feedback, mindset, academic motivation, culture, China, Finland

## Abstract

Given that little is known how peer feedback reflects adolescents’ academic well-being in different cultures, this study investigates, by means of multiple-group structural equation modeling (SEM), the influence of peer feedback on the mindset and academic motivation of Chinese (*N* = 992) and Finnish (*N* = 870) students in the fourth to the ninth school grades. Within this investigation, we also explore the culture-invariant and culture-dependent nature of student feedback, mindset and academic motivation. The results indicate that the way students praise their peers in their feedback primes and modifies their mindsets and academic motivation. Person-focused praise reflects a fixed mindset and negative academic motivation (i.e., avoidance), whereas process-focused praise undermines negative academic motivation. The pupils in the two samples had growth mindsets. However, the Finnish students preferred to bestow neutral praise and to be more negative with regard to their academic motivation whereas the Chinese students favored process- and person-focused praise, the former reflecting not only their growth mindset but also their positive academic motivation (i.e., trying).

## Introduction

This study explores the influence of peer feedback on the mindset and academic motivation of students in China and Finland. We argue that a growth mindset and academic motivation are key issues in school learning, and we aim to establish a multiple-group structural equation model (SEM) that reveals the culture-invariant and culture-dependent nature and influence of peer feedback on learning.

China and Finland represent two very different cultural and educational climates, although both have been successful in the Programme for International Student Assessment (PISA, OECD, 2019). Academic achievement and competition are major learning-related concerns in China (Ma et al., 2013), and numerous students take tutorials and attend special schools in order to be higher achievers academically. Given their effort-oriented culture (Dweck, 2000; Hofstede et al., 2010; Wang and Ng, 2012), Chinese students prefer to attribute learning achievement to effort rather than to ability (Zhang et al., 2019). On the other hand, individual holistic development and “equal opportunity and high-quality education for all citizens” (Finnish National Agency for Education, 2016) are highlighted more than pure academic achievement in Finland (Tirri and Kuusisto, 2013). In addition, Finland has been identified as a low-context culture (Hall, 1976), meaning that it is the content of the message that is the most important and not the way it is communicated. As a low-context culture, Finland favors neutral communication, and silence is seen as politeness. This characteristic also fits in well with the status of Finland as a neutral country between east and west, with a communication style that is explicit and neutral. In China, on the other hand, with its high-context culture and focus on style, the emphasis is on how the message is delivered (Hall, 1976). Most of the information is “either in the physical context or internalized in the person, while very little in the coded, explicit, transmitted part of the message” (p. 92). All this derives from the centuries-old, complex culture in China, familiarity with which is a prerequisite for understanding the message. Given the above-mentioned similarities and differences in the two cultures, and that human minds are culture-dependent (Gardner, 1999), we decided to investigate and compare students’ views on these issues and on the influence of peer feedback in China and Finland.

Studies over the last three decades have attested to the positive role of feedback in learning, especially in the performance of school learners (Hancock, 2002; Hattie, 2003; Gunderson et al., 2018). An increasing number of studies discuss the effect of positive feedback from parents and teachers on children’ self-esteem (Felson and Zielinski, 1989; Brummelman et al., 2014, 2017). The quality of the praise in feedback seems to be crucial, given that “the wrong kind of praise creates self-defeating behavior while the right kind motives students to learn” (Dweck, 2007, p. 34). For example, inflated praise has been found to predict lower self-esteem for most children, and to lead to higher levels of narcissism in some students (Brummelman et al., 2017). When educators issue praise without real substance or honesty, it is just empty words, “because it carries little information … and too often deflects attention from the task” (Hattie and Timperley, 2007, p. 96).

Moreover, children who have received person-focused praise from teachers tend to undermine their achievement and self-worth (Kamins and Dweck, 1999). There is also evidence that students who are praised for their intelligence do not want to do challenging tasks and cannot cope with setbacks in learning (Mueller and Dweck, 1998). Process-focused praise, on the other hand, allows for mistakes and challenges students to try harder (Mueller and Dweck, 1998; Kamins and Dweck, 1999). It also helps in the development of incremental beliefs, namely a growth mindset, whereas person-focused praise promotes entity beliefs, namely a fixed mindset (Mueller and Dweck, 1998; Kamins and Dweck, 1999; Dweck, 2006, 2017; Cimpian et al., 2007; Cimpian, 2010; Zentall and Morris, 2010). Furthermore, process-focused praise helps children to see human qualities as malleable, whereas person-focused praise encourages them to adopt more fixed views (Gunderson et al., 2013).

Other feedback styles such as neutral acknowledgment (Ferrar et al., 2019), outcome praise (Kamins and Dweck, 1999) and luck judgment (Butler, 1986, 2000) also affect cognitive and motivational development. According to Kamins and Dweck (1999), outcome praise helps to avoid negative responses in the face of setbacks, and neutral acknowledgment has also been found to promote individuals’ cognitive development and to motivate learning (Ferrar et al., 2019). Moreover, the impact of neutral feedback generally derives from the fact that “neutral acknowledgments may communicate to the child that their task behavior is worth attention, which could increase their interest in and motivation toward learning opportunities” (Ferrar et al., 2019, p. 376). In other words, neutral feedback conveys positive but mixed (i.e., ego-involved and task-involved) rather than clear and unidimensional messages (Butler, 1987). Even though the precise benefits of outcome praise and neutral acknowledgment are still not clear, previous studies have verified the positive role of neutral feedback, similar to that of process praise. Finally in this context, luck is an external factor that is not influenced by individuals, and it is like a fixed mindset the qualities of which are inherent and cannot be changed or developed by individuals. Luck is out of reach, and it happens when it happens. It has been described as a major determinant of learning performance, especially among pupils with a low-socio-economic status (Butler, 1986). Attributions of performance to luck seem to reflect “uncertainty about the causes of performances more than they do a generally fatalistic world view” (Butler, 2000, p. 275).

Gunderson et al. (2013, 2018) showed in their longitudinal studies that parental praise of toddlers predicted the children’s incremental beliefs 5 years later (Gunderson et al., 2013) and their academic achievement in the fourth grade (Gunderson et al., 2018). However, no direct relation between process praise and academic achievement was found in Gunderson et al.’s investigations, hence the present study focuses on the direct relationship. We adopted the multivariate multi-group approach that was successfully used by Yeager et al. (2011) in comparing the mindsets of Finnish and American students in the seventh-to-ninth grades, related to peer conflicts. According to Yeager et al. (2011) findings, a growth mindset reduced the desire for vengeance among students in both countries, indicating that enhancing awareness of a growth mindset and supporting such thinking radically enhances academic resilience and achievement (see also Aronson et al., 2002; Blackwell et al., 2007; Yeager et al., 2019). A growth mindset also tempers the negative effects of academic under-achievement, whereas poor school achievement is related to a fixed mindset (Claro et al., 2016). Similarly, higher achievement levels during challenging school transitions and better completion rates in demanding school courses have been reported among students with a growth mindset (Blackwell et al., 2007).

Regardless of feedback and mindset, students need academic motivation to study and to be engaged in their learning process. The academically gifted and talented may have low academic ambition and lack the motivation to do well in their studies (Garn and Jolly, 2014). In most cases, a lack of motivation among gifted students reflects an imbalance between what motivates them and the opportunities provided in the learning environment (Whitmore, 1986). Intervention strategies that are recommended to reverse the situation include boosting individuals’ self-concept, strengthening counseling services in the vicinity and creating inclusive learning environments in which students have more autonomy and freedom (Reis and McCoach, 2000).

The focus in the studies on feedback in learning described above is on parents and teachers as feedback givers and school pupils, especially preschool children, as the recipients, and little is known the extent to which peer feedback among adolescents affects their academic well-being. Despite the amount of existing research on the association between feedback and well-being in academic settings, a gap remains concerning how feedback affects the mindset and academic motivation of adolescents simultaneously. Moreover, there is a paucity of knowledge about the culture-dependent and culture-invariant nature of the above issues in two cultural settings. It is thus worthwhile investigating peer feedback and how it is given among school adolescents in different cultures (e.g., China and Finland). The present study also focuses on how this feedback affects the mindsets of these students and their academic motivation. We seek answers to the following research questions:

• RQ1: How do Chinese and Finnish students perceive the nature of feedback, mindset and academic motivation in learning?
• RQ2: How does the feedback that Chinese and Finnish students give to their peers reflect their (2a) mindsets and (2b) academic motivation in learning?

On the basis of earlier empirical findings, we hypothesis that the feedback students give to their peers reflects their mindsets and academic motivation. We assume, first, that person-focused praise is related to a fixed mindset whereas process-focused praise is related to a growth mindset (Hypothesis 1, see Dweck, 2006, 2017; Gunderson et al., 2013); and second, that person-focused praise is related to negative academic motivation whereas process-focused praise is related to positive academic motivation (Hypothesis 2, see Mueller and Dweck, 1998; Kamins and Dweck, 1999).

## Materials and Methods

### Participants and Procedure

Among the 1,862 participants (47.5% females, *M*_age_ = 12.9, *SD* = 1.675) were 992 Chinese (46.1% females, *M*_age_ = 13.2, *SD* = 1.602) and 870 Finnish (49.2% females, *M*_age_ = 12.6, *SD* = 1.714) fourth-to-ninth-grade adolescents from two Chinese and two Finnish public schools. The Chinese schools were located in Sichuan Province while the two Finnish schools were in Helsinki. There were more students from secondary schools than from elementary schools in both the Chinese (56.4%) and the Finnish (71.0%) samples.

The Chinese students’ grades (*M*_language_ = 7.5, *SD* = 0.832; *M*_math_ = 6.8, *SD* = 1.491) were based on standardized tests conducted in the autumn of 2017 and the spring of 2018, respectively, whereas the Finnish students’ grades (*M*_language_ = 8.1, *SD* = 1.228; *M*_math_ = 8.0, *SD* = 1.490) reflected the teachers’ assessment of examinations they had designed and the students’ classroom activities in the autumn of 2017 and the spring of 2018, respectively. Most Finnish grades were on a scale ranging from 4 to 10 (4 = fail, 5 = passable, 10 = excellent). To ensure uniformity in this study we converted the original Chinese grade scale (0–100, < 60 = fail, 60 = lowest passing score, 100 = full score) and partial Finnish grades (0–4, 0 = fail, 1 = passable, 4 = excellent) to the common Finnish scale of 4 to 10 through data weighting. Overall, the students seemed to achieve higher grades in languages (*M* = 7.8, *SD* = 1.077) than in mathematics (*M* = 7.3, *SD* = 1.599).

Consent for participation among the Chinese sample was given by the school principals and the students’ parents. The first author was present to explain the procedure when the Chinese students were organized by their headteachers and completed the written questionnaires in their classrooms. Permission for the participation of the Finnish sample was granted by the City of Helsinki, the schools’ administrative committees and the students’ parents. The Finnish students were informed about the procedure by their teachers beforehand, and all the Finnish data were gathered by means of Qualtrics software. The students completed the survey during school hours under teacher supervision, and their grades were obtained from the respective administration offices in all four schools.

### Measurement Instruments

#### Feedback

The giving of feedback was investigated in this study via the concept of praise (Gunderson et al., 2013). The participants were asked to grade 16 items on a five-point scale (1 = strongly disagree, 5 = strongly agree) in terms of how they praised their classmates for the achievement of good grades in their learning. In accordance with the recommendations of Gunderson et al. (2013) and based on confirmatory factor analysis (CFA, conducted by the authors of this paper), the instrument was divided into three factors [Chinese: χ^2^(83) = 222.560, CFI = 0.946, TLI = 0.921, RMSEA = 0.046, SRMR = 0.040; Finnish: χ^2^(87) = 393.528, CFI = 0.950, TLI = 0.931, RMSEA = 0.071, SRMR = 0.049]: *neutral praise* (e.g., “Fine result,” “Great!”), *person praise* (e.g., “You are so gifted,” “You were really lucky!”) and *process praise* (e.g., “You must have worked hard to achieve this score,” “It was worthwhile reading for the exam!”). Equally noteworthy was that, in the present study, *neutral praise* included both neutral acknowledgment (Ferrar et al., 2019) and outcome praise (Kamins and Dweck, 1999), and *person praise* included luck (Butler, 1986, 2000). All the items are presented in the Supplementary Appendix.

#### Mindset

The instruments derived from both the implicit theory of intelligence (ITI, Dweck, 2000) and the implicit theory of giftedness (ITG, Dweck, 2000; Laine et al., 2016; Zhang et al., 2017) included four items measuring the nature of human qualities, based on a six-point scale (1 = strongly agree, 6 = strongly disagree). According to the CFA, the mindset model indicated a good fit within a two-factor framework [Chinese: χ^2^(14) = 45.272, CFI = 0.981, TLI = 0.963, RMSEA = 0.054, SRMR = 0.024; Finnish: χ^2^(14) = 25.424, CFI = 0.996, TLI = 0.993, RMSEA = 0.037, SRMR = 0.014]: the *implicit theory of intelligence* (e.g., “You have a certain amount of intelligence, and you really can’t do much to change it”) and the *implicit theory of giftedness* (e.g., “You have a certain amount of giftedness, and you really can’t do much to change it”). Among these scales, values equal to or above 3.5 indicate a growth mindset (intelligence or giftedness can change, it is malleable) whereas values less than 3.5 indicate a fixed mindset (intelligence or giftedness cannot change, it is fixed).

#### Academic Motivation (AM)

The Academic Motivation (Campbell et al., 2018) instrument consisted of 11 items rated on a five-point scale (1 = strongly disagree, 5 = strongly agree) and measuring study engagement and motivation. Both previous empirical evidence and the CFA supported a two-factor structure [Chinese: χ^2^(38) = 79.658, CFI = 0.970, TLI = 0.956, RMSEA = 0.037, SRMR = 0.028; Finnish: χ^2^(38) = 143.585, CFI = 0.942, TLI = 0.916, RMSEA = 0.064, SRMR = 0.046]: *trying* (e.g., “I have a strong interest in solving problems”) and *avoidance* (e.g., “Few things taught at school interest me”).

In sum, the value of fit indices for all three instruments (χ^2^/Df = 1.82–4.52, CFI = 0.942–0.996, TLI = 0.916–0.993, RMSEA = 0.037–0.071, SRMR = 0.014–0.049) in this study were satisfactory, with the criterion of around three on the chi-square and degree-of-freedom ratios, above 0.90 on the comparative fit index (CFI) and the Tucker-Lewis index (TLI), and below 0.08 on the root mean square error of approximation (RMSEA) and the standardized root mean residual (SRMR) (Chen, 2014). These values indicate sufficient structural validity for further path-coefficient analysis.

### Statistical Analysis

Similar methods were applied in the analyses of the Chinese and Finnish samples. First, the IBM Statistical Package for Social Sciences (SPSS) version 24 was used to screen for missing values: there were none (0.0%) in the Chinese sample and very few (1.6%) in the Finnish sample. According to Little’s MCAR test, the data were missing completely at random (China: no EM estimated statistics given that there were no missing values; Finland: χ^2^(32) = 41.010, *p* = 0.132).

Second, previous theoretical underpinnings concerning the three instruments preliminarily identified the latent factors for model construction. The next step was to conduct a confirmatory factor analysis (CFA) with R (R Core Team, 2013) to test the fit of the indices across the three separate measurement models (1. praise; 2. mindset; 3. academic motivation). After this, another CFA was conducted to combine the three separate measurement models in a full mediational model.

Third, to establish a more robust mediational model we tested configural, metric and scalar measurement invariance step by step to ensure the psychometric equivalence of the construct factor patterns, the factor loadings and the item intercepts across the Chinese and the Finnish groups (Putnick and Bornstein, 2016). In our data, configural measurement invariance referred to the invariant factor structure across both Chinese and Finnish students, metric invariance referred to the identical response tendencies of the Chinese and Finnish students, and scalar invariance referred to the comparable response means across both student samples. As Table 1 shows, although the metric invariance test was significant (*p* < 0.001), the changed model fit indices were small (ΔCFI = 0.004, ΔTLI = 0.003 < 0.010; ΔRMSEA = 0.001, ΔSRMR = 0.003 < 0.005, see Chen, 2007; Fan and Sivo, 2009; Putnick and Bornstein, 2016). Thus, metric invariance, namely factor loadings of the same model across distinct groups, was supported. However, the scalar invariance test was significant, and the change in the fit indices was beyond the acceptable scope (ΔCFI = 0.031, ΔTLI = 0.033 > 0.010; ΔRMSEA = 0.008, ΔSRMR = 0.006 > 0.005). Merging the two samples was not a suitable option because the item-intercept invariance was not supported. Accordingly, we constructed a multi-group mediational model by country in which the factor loadings were set to be invariant.

**TABLE 1:** Measurement invariance across the Chinese and Finnish samples^a^.

	Df (ΔDf)	χ^2^ (Δχ^2^)	Pr(>χ^2^)	CFI (ΔCFI)	TLI (ΔTLI)	RMSEA (ΔRMSEA)	SRMR (ΔSRMR)
Configural	1014	2144.038		0.942	0.932	0.037	0.051
Metric	1042 (28)	2247.171 (103.66)	<0.001	0.938 (−0.004)	0.929 (−0.003)	0.038 (0.001)	0.054 (0.003)
Scalar	1070 (28)	2899.867 (746.49)	<0.001	0.907 (−0.031)	0.896 (−0.033)	0.046 (0.008)	0.060 (0.006)

*Δ = differences in fit indices. ^a^Method = satorra.bentler.2001.*

Fourth, a multiple-group structural equation model was established using the lavaan of R package (Rosseel, 2012). First, the mediational model was estimated without control variables, then the students’ class degree and maths grades were added as covariates to assess the extent to which these variables affect each factor and the path coefficients. Our first aim was to find out how students perceived the nature of feedback, mindset and academic motivation in learning (RQ1). Second, we were interested in whether the variation in mindset could be attributed to the feedback scale, and if so to what extent (RQ2a), and in the predictive value of the mindset in terms of academic motivation (RQ2b).

In general, we used the robust maximum likelihood (MLR) estimator because the measurements were ordinal (with no less than five response options) and symmetrically distributed (Raykov, 2012), and full information maximum likelihood (FIML) to estimate the missing data.

## Results

### RQ1: How Do Chinese and Finnish Students Perceive the Nature of Feedback, Mindset, and Academic Motivation in Learning?

The Supplementary Appendix shows the comparisons between the mean values of the Chinese and Finnish students in the 35 items of the survey (Praise: PRAISE1 – PRAISE16, Mindset: ITI1 – ITI4 and ITG1 – ITG4, Academic motivation: AM1– AM11). The results of the independent-samples t-test revealed statistically significant differences in 23 (out of 35) survey items in the two sets of responses. In terms of praise, the Chinese students tended to be more process- (PRAISE3, 7, 15, *p* < 0.001, see Supplementary Appendix) and person- (PRAISE 2, 4, 6, 8, 14, *p* ≤ 0.019, see the Supplementary Appendix) oriented, whereas the Finnish students were more neutral (PRAISE1, 5, 9, 13, *p* ≤ 0.023, see the Supplementary Appendix). On the mindset question, the overall response tendency for all eight items was equal to or above the average value of 3.5 (*M* = 3.5–4.2, *p* < 0.001), indicating that all the participants favored a growth over a fixed mindset. However, the average values of the Chinese students on items ITG3 [*M*_Ch_ = 4.2, *SD* = 1.411; *M*_Fin_ = 3.7, *SD* = 1.544; *t*(1766.375) = −6.183, *p* < 0.001] and ITG4 [*M*_Ch_ = 4.0, *SD* = 1.532; *M*_Fin_ = 3.6, *SD* = 1.529; *t*(1856) = −5.688, *p* < 0.001] were statistically significantly higher than those of the Finnish students, indicating that they had more malleable views about the developmental potential of giftedness. In terms of academic motivation, there was a stronger tendency toward the negative, namely avoidance, than the positive among the Finnish students (AM4, 7–11, *p* ≤ 0.001, see the Supplementary Appendix).

In sum, we conclude from the item-level findings related to the first research question that: (1) Chinese students show a preference for process-focused and person-focused praise with regard to their peers, whereas Finnish students prefer to use neutral praise; (2) both the Chinese and the Finnish students had growth mindsets, but the Chinese students’ views on the developmental potential of giftedness were more malleable than those of the Finnish students; (3) the Finnish students reported more avoidance-oriented academic motivation.

Table 2 presents the correlations of all the latent factors on mindset, praise and academic motivation among the Chinese and the Finnish samples. First, both ITI and ITG were associated negatively with person-focused praise (ITI: β = −0.205 to −0.212, *p* < 0.001; ITG: β = 0.144–0.162, *p* ≤ 0.004) and avoidance (ITI: β = −0.150 to −0.325, *p* ≤ 0.005; ITG: β = −0.110 to −0.321, *p* ≤ 0.023). Second, all forms of praise correlated positively with trying (β = 0.225–0.526, *p* < 0.001), whereas neutral and process praise were associated negatively with avoidance (β = −0.133 to −0.367, *p* ≤ 0.009). Regarding the inner correlations of each measure, ITI and ITG (β = 0.634–0.914, *p* < 0.001), neutral, person-related and process praise (β = 0.578–0.861, *p* < 0.001) tended to correlate highly.

**TABLE 2:** Correlations of the latent factors among the Chinese and Finnish samples.

	Neutral Chinese (Finnish)	Person Chinese (Finnish)	Process Chinese (Finnish)	ITI Chinese (Finnish)	ITG Chinese (Finnish)	Trying Chinese (Finnish)	Avoiding Chinese (Finnish)
Neutral	1						
Person	0.810***(0.591***)	1					
Process	0.696***(0.663***)	0.578***(0.861***)	1				
ITI	−0.034(0.059)	−0.205***(−0.212***)	0.073(−0.100*)	1			
ITG	0.011 (0.009)	−0.144**(−0.162***)	0.139**(−0.090*)	0.914***(0.634***)	1		
Trying	0.407***(0.291***)	0.335***(0.225***)	0.526***(0.278***)	0.211**(0.032)	0.251**(−0.066)	1	
Avoiding	−0.169**(−0.197***)	0.080 (0.093)	−0.367***(−0.133**)	−0.325***(−0.150**)	−0.321***(−0.110*)	−0.353***(−0.129*)	1

*p < 0.05, **p < 0.01, ***p < 0.001.

### RQ2: How Does the Feedback That Chinese and Finnish Students Give to Their Peers Reflect Their (2a) Mindsets and (2b) Academic Motivation in Learning?^1^

**FIGURE 1:**
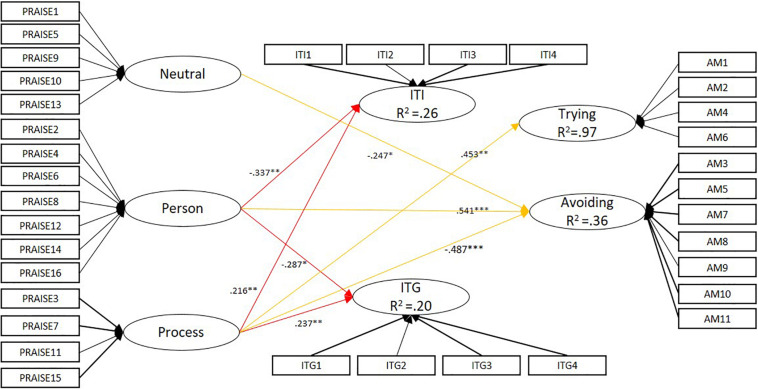
The Chinese mediational model: Praise – Mindset – Academic motivation. ^∗^*p* < 0.05, ^∗∗^*p* < 0.01, ^∗∗∗^*p* < 0.001. The thicker lines in the measurement model represent factor loadings > 0.7, the narrower lines < 0.7.

**FIGURE 2:**
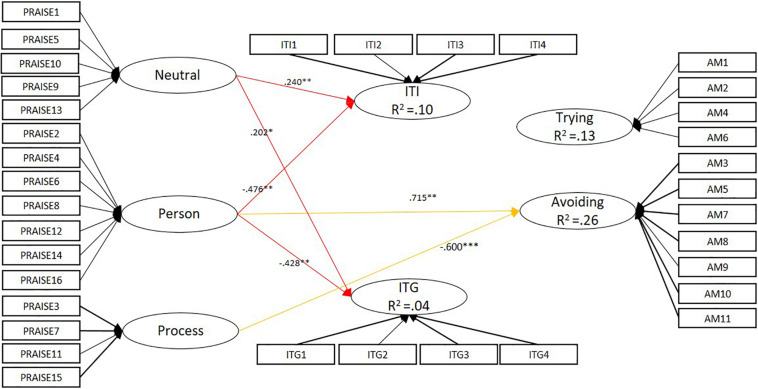
The Finnish mediational model: Praise – Mindset – Academic motivation. ^∗^*p* < 0.05, ^∗∗^*p* < 0.01, ^∗∗∗^*p* < 0.001. The thicker lines in the measurement model represent factor loadings > 0.7, the narrower lines < 0.7.

In accordance with the preliminary analysis (measurement models and measurement invariance), we established a multi-group mediational model taking the country as the group classification: the fit indices [χ^2^(1152) = 2577.526, CFI = 0.927, TLI = 0.916, RMSEA = 0.040, SRMR = 0.050] were acceptable. Figures 1, 2 show the parameters of the SEM regressions of praise on mindset and academic motivation, and mindset on academic motivation in the Chinese and Finnish samples.

With regard to mindset, a negative relation was detected in both samples between person-focused praise and implicit theories of intelligence (β_Ch_ = −0.337, *p* = 0.003; β_Fin_ = −0.476, *p* = *0.004*) and giftedness (β_Ch_ = −0.287, *p* = *0.018*; β_Fin_ = −0.428, *p* = 0.009). However, among the Chinese students process-related praise reflected a growth mindset regarding intelligence (β = 0.216, *p* = 0.005) and giftedness (β = 0.237, *p* = 0.004), which was not the case in the Finnish sample. Among the Finnish students, neutral praise reflected a growth mindset regarding intelligence (β = 0.240, *p* = 0.002) and giftedness (β = 0.202, *p* = 0.010), which was not indicated among their Chinese counterparts.

In sum, all three ways of giving feedback (neutral, person-focused, process-focused) were statistically related to the students’ views on the malleability of their giftedness and intelligence. Person-focused praise was significantly related to a fixed mindset across both samples, whereas process-focused praise was related to a growth mindset in the Chinese case. These results support Hypothesis 1. Equally noteworthy is that neutral praise was found related to a growth mindset among Finnish adolescents.

Similarly, with regard to academic motivation the higher the usage of person-focused praise, the higher the likelihood of avoidance-oriented motivation (β = 0.54–0.72, *p* ≤ 0.001). Furthermore, process-focused praise negatively reflected avoidance-oriented motivation (β_Ch_ = −0.49, β_Fin_ = −0.60, *p* < 0.001). However, a similar predictive function was rather stronger in the Finnish sample than among the Chinese students. On the other hand, neutral praise was found to have a negative effect on avoidance-oriented motivation among Chinese students (β = 0.25, *p* = 0.043). In addition, process-focused praise was found to prompt trying-oriented motivation among the Chinese sample (β = 0.45, *p* = 0.001), which was not specified in the Finnish sample.

These findings imply that the way in which students praise their peers could reflect their academic motivation. Person-focused praise turned out to be positively related and process-focused praise to be negatively related to avoidance-oriented motivation in all cases. Furthermore, process-focused praise was found to reflect students’ trying-oriented motivation in the Chinese sample. As far as the second hypothesis was concerned, it turned out that negative academic motivation (i.e., avoidance) in students was reflected in the use of person-focused praise, but was undermined in process-focused praise.

## Discussion

In the present study, we investigated the influence of peer feedback on the mindset and academic motivation of Chinese and Finnish students in the fourth to the ninth school grades. Our first research question explored how students perceived the nature of feedback, mindset and academic motivation in learning. The second research question investigated how the feedback students gave to their peers related to their mindset and academic motivation by means of a multiple-group structural equation model (SEM). In line with earlier research findings, the second question focused on two issues: first, is the feedback related to the students’ mindset in terms of learning (Hypothesis 1), and second, is it related to their academic motivation (Hypothesis 2)?

### The Culture-Variant and Culture-Dependent Nature of Feedback, Mindset, and Academic Motivation

The Chinese students used both process- and person-focused praise in giving feedback to their peers, whereas the Finnish students were mainly neutral in their praising style, reflecting the different cultural values in education. Chinese philosophy emphasizes both effort and luck, or more precisely destiny. Common sayings in China, for instance, highlight either effort – “Making an effort to compensate for inadequate intelligence” (qín néng bǔ zhuō) – or power that is beyond one’s control – “Man proposes, God disposes” (móu shì zài rén, chéng shì zài tiān). The former saying reflects the Chinese effort-oriented culture (Dweck, 2000; Hofstede et al., 2010; Wang and Ng, 2012), in which students attribute individual performance to effort rather than ability (Zhang et al., 2019). Particularly noteworthy is that destiny in Chinese philosophy is not fixed: it has the characteristic of constant motion and regular recurrence (Raphals, 2003). The results related to Finland could be attributable to the low-context communication style with its short and direct messages, and reflect recent findings that both neutral and process-focused praise have similar effects in promoting cognitive and motivational development (Ferrar et al., 2019).

The findings of growth-oriented mindset among all adolescents and the higher malleability attribution to giftedness in Chinese students than their Finnish counterparts, confirms results reported in previous empirical studies on China and Finland well (Zhang et al., 2017, 2019). The Finnish students tended to report more negative academic motivation, which is reflected in the latest PISA studies, Finland having lost its top position and China having taken the lead (OECD, 2019).

### The Culture-Variant and Culture-Dependent Relations Between Feedback, Mindset, and Academic Motivation

Taking the relations together, culture-invariant paths were found in the Chinese and Finnish models: the more the students bestowed person-focused praise, the more likely they were to have a fixed mindset and negative academic motivation, indicating a lack of willingness to put effort into their learning. This finding is in accordance with previous research results indicating that children who are praised for their ability tend to identify intelligence as a fixed trait (Mueller and Dweck, 1998; Zentall and Morris, 2010), and that the praising of intelligence has negative consequences in terms of student motivation (Mueller and Dweck, 1998; Cimpian, 2010; Zentall and Morris, 2010; Gunderson et al., 2013). Furthermore, students whose praise was more process-focused were less likely to show negative academic motivation. These similarities could imply that the way students praise their peers primes and modifies their mindsets and motivation. However, we did not find a path from mindset to academic motivation in either sample in the present study. Similar results have been reported in other studies, which is why Dweck’s theory has been somewhat criticized (Leondari and Gialamas, 2002; Dupeyrat and Mariné, 2005).

In terms of culture-dependent relations, the Chinese students who bestowed process-focused praise were more likely to have a growth mindset and to show positive academic motivation. We also found a direct path from process-focused praise to positive academic motivation, in contrast to the indirect path Gunderson et al. (2018) identified. In general, the Chinese model reflects Dweck’s theory (2000) and Gunderson et al. (2013) empirical study. The same connection was not evident among the Finnish students: their neutral praise rather reflected their growth mindset, but neutral praise was not associated with academic motivation. This is in accordance with previous findings that neutral feedback, similar to process-focused praise, plays a positive role in promoting cognitive development in learning because it yields enhancing information to the recipients (Ferrar et al., 2019), as outcome praise does in the present study. However, the lack of an association between praising and positive academic motivation implies that the notions of lifelong learning and process orientation set out in the *National Core Curriculum for Basic Education 2014* (Finnish National Agency for Education, 2016) are not yet established in Finnish schools.

It is worth pointing out that the present research is a comparative study. We investigate how feedback is given, and how this feedback relates to mindset and academic motivation by comparing Chinese and Finnish adolescents. Our aim was to explore the similarities and differences in the results and explain them from the perspective of cultural values and educational views in China and Finland. This is why we refer to culture-invariant nature instead of similarity, and culture-dependent nature instead of difference. Cultural values and educational views are the main concerns, but they are not the mere factors. There should be other factors behind the results described above.

### Methodological Reflections

The present study has several limitations. The first one relates to the samples. The Finnish schools were located in urban areas and the Chinese schools in a rural area. The results could have been different had the samples represented equivalent locations. However, Finnish and Chinese cultures are quite different, making identical samples difficult to find. In addition, we should point out that our Chinese sample might give an even better view of average Chinese schools than the PISA samples collected from the academically highest achieving schools in Beijing and Shanghai. Nevertheless, given that the samples in the present study are limited to Chinese and Finnish schools, the validity of the results is somewhat limited. Hence, similar and diverse samples are needed to enable the findings to be further generalized. The second limitation relates to the multiple-group mediational model. The different scales used in the study had different numbers of items, which could have led to inaccuracy in the correlates and aggregation of the relevant factors (Ketonen et al., 2018). The data in both countries were collected cross-sectionally, which limits the interpretation of the mediational model. A longitudinal study is needed to elucidate the interconnected differences and relationships among the scaling factors. Third, the data was largely self-reported, which would cause measurement discrepancies from real-situational results (Hietajärvi et al., 2019). For example, Chinese and Finnish students might rate the scales differently, and Chinese students might be more lenient than Finnish students. Therefore, additional data sources such as the evaluations of relevant educators (e.g., parents or teachers) would supplement the findings.

### Implications for Education

The present study has several strengths that have implications for education. To promote a growth mindset in students in terms of learning and high academic motivation, parents and teachers should encourage them to give process-focused feedback to their peers. Practice in giving peer feedback should be included in courses at all grade levels in schools. By implication, therefore, teacher education should also include training in how to guide students to give constructive feedback to their peers, and to help them understand how process-focused feedback modifies mindsets and primes academic motivation in learning. Thus, it is not enough merely to teach mindset theory in schools or teacher education. The implementation of growth-mindset pedagogy (Rissanen et al., 2019) requires educational interventions in which students are taught how to provide peer feedback that promotes a growth mindset and academic motivation.

## Data Availability Statement

The raw data supporting the conclusions of this article will be made available by the authors, without undue reservation, to any qualified researcher.

## Ethics Statement

The study involving human participants was reviewed and approved by the University of Helsinki Ethical Review Board in the Humanities and Social and Behavioural Sciences. Written informed consent to participate in this study was provided by the participants’ legal guardian/next of kin.

## Author Contributions

JZ designed the study, conducted the analysis, interpreted the results, and drafted the manuscript. EK designed the study, interpreted the results and discussion. PN revised the analysis and the result. KT designed the study, interpreted the discussion, and established the CoPErNicus project. All authors reviewed and revised the manuscript and approved the submitted version.

## Conflict of Interest

The authors declare that the research was conducted in the absence of any commercial or financial relationships that could be construed as a potential conflict of interest.

## Footnotes

1 The respective Chinese and Finnish mediational models were specified including class level and maths grade as covariates. Similarly, across both samples, students with higher marks in maths tended to give more neutral praise (β = 0.098–0.127, *p* ≤ 0.011), to keep a growth mindset (β = 0.143–0.343, *p* ≤ 0.001) and to adopt more trying-oriented (β = 0.208–0.620, *p* < 0.001) or less avoidance-oriented motivation (β = −0.170 to −0.195, *p* < 0.001). In addition, students in higher-level classes were more likely to display a growth mindset with regard to intelligence (β = 0.091–0.405, *p* ≤ 0.020) and to bestow less process-focused praise (β = −0.142 to −0.198, *p* ≤ 0.001). On the other hand, among the Chinese students only, the higher level the class was, the stronger academic motivation they showed (β = 0.467, *p* < 0.001); the higher maths marks they achieved, the more process-focused praise they bestowed (β = 0.122, *p* = 0.005). Furthermore, among the Finnish students only, there were negative correlations between class level and neutral (β = −0.172, *p* < 0.001) praise, and between maths marks and person-focused praise (β = −0.158, *p* < 0.001).
