# Supplementary material for: Peer Feedback Reflects the Mindset and Academic Motivation of Learners
Source: Front Psychol. 2020 Jul 16;11:1701. doi: 10.3389/fpsyg.2020.01701 (PMC7378527; doi:10.3389/fpsyg.2020.01701)
Supplement: Supplementary file 1 [file Table_1.DOCX]

# Appendix

| Independent Sample T-Test | | | | | |  |
| --- | --- | --- | --- | --- | --- | --- |
|  | China  M (SD) | Finland  M (SD) | | t value | p | |
| *Praise* (16 items) | | | | | |  |
| *Neutral* |  |  |  | |  | |
| PRAISE1 ‘Wow! That’s a really good score!’ | 3.1(1.139) | 3.3(1.190) | 2.28 | | 0.023* | |
| PRAISE5 ‘It went well.’ | 3.3(1.297) | 3.5(1.284) | 3.29 | | 0.001** | |
| PRAISE9 ‘Fine result!’ | 3.0(1.190) | 3.7(1.202) | 12.08 | | <0.001*** | |
| PRAISE10 ‘You always do great!’ | 3.0(1.241) | 3.1(1.287) | 1.61 | | 0.107 | |
| PRAISE13 ‘Great!’ | 2.9(1.327) | 3.7(1.174) | 14.94 | | <0.001*** | |
| *Person* |  |  |  | |  | |
| PRAISE2 ‘You are so smart in this subject!’ | 3.2(1.207) | 2.9(1.174) | -5.23 | | <0.001*** | |
| PRAISE4 ‘Here you see, sometimes things just go well!’ | 2.9(1.287) | 2.7(1.253) | -2.34 | | 0.019* | |
| PRAISE6 ‘You are so gifted!’ | 3.1(1.202) | 2.8(1.250) | -4.49 | | <0.001*** | |
| PRAISE8 ‘The exam questions seemed to be suitable for you!’ | 2.8(1.248) | 2.5(1.287) | -4.48 | | <0.001*** | |
| PRAISE12 ‘Well done. I guess you guessed that these questions would be asked.’ | 2.4(1.228) | 2.3(1.269) | -1.02 | | 0.31 | |
| PRAISE14 ‘You were born to be successful!’ | 2.4(1.236) | 2.2(1.232) | -3.07 | | 0.002** | |
| PRAISE16 ‘You were really lucky!’ | 2.4(1.324) | 2.4(1.370) | -0.51 | | 0.61 | |
| *Process* |  |  |  | |  | |
| PRAISE3 ‘You must have worked hard to achieve this score!’ | 3.6(1.243) | 2.8(1.287) | -13.28 | | <0.001*** | |
| PRAISE7 ‘I can see that you have put a lot of effort into learning new issues.’ | 3.5(1.217) | 2.8(1.293) | -11.77 | | <0.001*** | |
| PRAISE11 ‘It was worthwhile reading for the exam!’ | 3.3(1.275) | 3.2(1.327) | -1.53 | | 0.125 | |
| PRAISE15 ‘You practised a lot and it can be seen in this result!’ | 3.5(1.194) | 2.8(1.327) | -11.24 | | <0.001*** | |
| *Mindset* (8 items) |  |  |  | |  | |
| *Implicit theory of intelligence (ITI)*  ITI1 ‘You have a certain amount of intelligence, and you really can’t do much to change it.’ | 4.2(1.402) | 4.1(1.518) | -1.51 | | 0.131 | |
| ITI2 ‘Your intelligence is something that you can’t change very much’ | 3.7(1.332) | 3.9(1.390) | 2.86 | | 0.004** | |
| ITI3 ‘To be honest, you can’t really change how intelligent you are.’ | 4.2(1.447) | 4.2(1.503) | 0.10 | | 0.920 | |
| ITI4 ‘You can learn new things, but you can’t really change your basic intelligence.’ | 4.0(1.516) | 3.9(1.468) | -1.13 | | 0.259 | |
| *Implicit theory of giftedness (ITG)*  ITG1 ‘You have a certain amount of giftedness, and you really can’t do much to change it.’ | 3.8(1.435) | 3.7(1.542) | -0.95 | | 0.339 | |
| ITG2 ‘Your giftedness is something that you can’t change very much.’ | 3.5(1.323) | 3.5(1.469) | -0.16 | | 0.873 | |
| ITG3 ‘To be honest, you can’t really change how gifted you are.’ | 4.2(1.411) | 3.7(1.544) | -6.18 | | <0.001*** | |
| ITG4 ‘You can learn new things, but you can’t really change your basic giftedness.’ | 4.0(1.532) | 3.6(1.529) | -5.69 | | <0.001*** | |
| *Academic motivation* (11 items) |  |  |  | |  | |
| *Trying* |  |  |  | |  | |
| AM1 ‘I have a strong interest in solving problems.’ | 3.2(0.980) | 3.2(1.098) | 0.32 | | 0.746 | |
| AM2 ‘When I don’t understand something right away, I still try to figure it out for myself.’ | 3.5(0.977) | 3.7(0.935) | 3.80 | | <0.001*** | |
| AM4 ‘I feel comfortable when I try out new ideas for solving problems.’ | 3.6(1.086) | 3.3(1.049) | -6.40 | | <0.001*** | |
| AM6 ‘I work hard and usually solve difficult problems by myself.’ | 3.4(0.926) | 3.4(0.978) | 0.09 | | 0.924 | |
| *Avoidance* |  |  |  | |  | |
| AM3 ‘Why work when you don’t have the ability?’ | 2.1(1.075) | 2.1(1.117) | 0.56 | | 0.579 | |
| AM5 ‘I will not work if I do not like the teacher.’ | 2.0(1.071) | 2.2(1.147) | 3.48 | | 0.001** | |
| AM7 ‘I do not work hard when I’m not interested.’ | 2.3(1.185) | 2.6(1.142) | 5.93 | | <0.001*** | |
| AM8 ‘I do not need help from the teacher to do well in school.’ | 1.8(0.952) | 2.5(1.029) | 13.57 | | <0.001*** | |
| AM9 ‘Few things taught at school interest me.’ | 1.9(0.956) | 2.5(1.113) | 14.12 | | <0.001*** | |
| AM10 ‘There are some things you won’t do well no matter how hard you try.’ | 2.6(1.194) | 3.0(1.101) | 7.38 | | <0.001*** | |
| AM11 ‘I can’t wait to get out of school each day.’ | 2.1(1.102) | 3.1(1.260) | 17.87 | | <0.001*** | |

**p* < 0.05, ***p* < 0.01, ****p* < 0.001.
